# *Epidermal growth factor receptor* (*EGFR*) mutation and personalized therapy in advanced nonsmall cell lung cancer (NSCLC)

**DOI:** 10.1007/s11523-013-0258-9

**Published:** 2013-01-30

**Authors:** Kunihiko Kobayashi, Koichi Hagiwara

**Affiliations:** Saitama Medical University, Moroyama, Japan

**Keywords:** Nonsmall cell lung cancer (NSCLC), *EGFR* mutation, EGFR-TKI, Gefitinib, Erlotinib

## Abstract

Before 2009, nonsmall cell lung cancer (NSCLC) was one disease entity treated by cytotoxic chemotherapy that provided a response rate of 20–35 % and a median survival time (MST) of 10–12 months. In 2004, it was found that activated mutations of the *epidermal growth factor receptor* (*EGFR*) gene were present in a subset of NSCLC and that tumors with *EGFR* mutations were highly sensitive to EGFR tyrosine kinase inhibitors (TKI). Four phase III studies (North East Japan (NEJ) 002, West Japan Thoracic Oncology Group (WJTOG) 3405, OPTIMAL, and EUROTAC) prospectively compared TKI (gefitinib or erlotinib) with cytotoxic chemotherapy as first-line therapy in *EGFR*-mutated NSCLC. These studies confirmed that progression-free survival (PFS) with TKIs (as the primary endpoint) was significantly longer than that with standard chemotherapy (hazard ratio [HR] = 0.16–0.49) from 2009 to 2011. Although the NEJ 002 study showed identical overall survival (OS) between the arms (HR = 0.89), quality of life (QoL) was maintained much longer in patients treated with gefitinib. In conclusion, TKI should be considered as the standard first-line therapy in advanced *EGFR*-mutated NSCLC. Since 2009, a new step has been introduced in the treatment algorithm for advanced NSCLC.

## Introduction

Recent sequencing of DNA to identify polymorphisms has catalyzed the quest for protein kinase “driver” mutations, which contribute to the transformation of a normal cell to a proliferating cancerous cell. On the other hand, kinase “passenger” mutations are considered to reflect mutations that merely build up in the course of cancerous cell replication and proliferation. At present, there are driver mutations in nonsmall cell lung cancer (NSCLC), such as *epidermal growth factor receptor* (*EGFR*) mutations [1–3], a fusion gene between echinoderm microtubule-associated protein-like 4 (*EML4*) and the anaplastic lymphoma kinase (*ALK*) [4, 5], and fusion genes with RET proto-oncogene (*RET*) [6–8], for which specific agents have been developed. In this manuscript, a road to personalized therapy by *EGFR* mutations in advanced NSCLC, which was the first experience to treat advanced NSCLC patients individually, is reviewed.

## Personalized therapy by *EGFR* mutations in advanced NSCLC

Dysregulation of protein kinases is frequently observed in cancer cells; therefore, protein kinases are attractive targets in the development of anticancer drugs. Small molecule inhibitors that block binding of adenosine-5′-triphosphate (ATP) to the tyrosine kinase catalytic domain have been developed, and gefitinib and erlotinib are the first generation of such agents, which act as tyrosine kinase inhibitors (TKI) at the *EGFR*. In 2004, three groups of researchers reported that activating mutations of *EGFR* detected by direct sequencing were present in a subset of NSCLC and that tumors with *EGFR* mutations were highly sensitive to EGFR-TKI [1–3].

Although this knowledge is the first evidence for division of subpopulations in NSCLC and of the possibility of treating NSCLC patients individually, there have been two streams of clinical studies. Clinical efficacy of EGFR-TKIs such as gefitinib or erlotinib has been investigated initially in unselected patients [9–13] and, subsequently, on the basis of clinical characteristics [14]. On the other hand, in order to develop personalized therapy in NSCLC, clinical efficacy of EGFR-TKIs has been indicated by molecular selection in phase 3 trials of NSCLC (Table 1) [15–19].

**Table 1:** Clinical studies using EGFR-TKI

	Second-line treatment	First-line treatment
Unselected patients	BR.21	
	ISEL	
	INTEREST	
	V-15-32	
Selection by background		IPASS
Selection by EGFR mutation		NEJ Gefitinib Study-02
		WJTOG 3405
		OPTIMAL (CTONG 0802)
		EURTAC-SLCG GECP06/01

### Unselected patients

In the BR.21 phase III comparative study [9], 731 previously treated NSCLC patients (unselected by *EGFR* mutations) were allocated randomly to the erlotinib or placebo groups at a ratio of 2:1. At the primary endpoints, erlotinib was significantly superior in terms of both progression-free survival (PFS) (2.2 months vs. 1.8 months, respectively, hazard ratio (HR) = 0.61, *p* < 0.001) and median survival time (MST) (6.7 months vs. 4.7 months, respectively, HR = 0.70, *p* < 0.001). On the basis of the results of BR.21, erlotinib has become a standard therapy for previously treated patients with advanced NSCLC and is now used in previously treated cases of NSCLC that may or may not have *EGFR* mutations.

In order to evaluate gefitinib, a phase III study (Iressa Survival Evaluation in Advanced Lung Cancer (ISEL)) was carried out [10]. A total of 1,692 patients refractory to or intolerant of their latest chemotherapy were randomized to receive either gefitinib (250 mg/day) or placebo plus best supportive care (BSC). The primary endpoint, MST, was 5.1 months in the placebo group and 5.6 months in the gefitinib group, with no significant differences between the two groups (*p* = 0.087). Therefore, efficacy of gefitinib in NSCLC patients unselected by *EGFR* mutations was not indicated. Another randomized phase III study (INTEREST) [11] compared gefitinib with standard second-line chemotherapy using docetaxel in 1,433 previously treated NSCLC patients unselected by *EGFR* mutations. As to overall survival (OS), which was the primary endpoint of the study, the HR was 1.020 (95 % confidence interval [CI]: 0.905–1.150) and did not exceed the preset upper limit (1.154), thus endorsing the noninferiority of gefitinib to docetaxel. However, the V-15-32 randomized phase III study, which aimed to confirm the noninferiority of gefitinib to docetaxel in regard to OS [12], was carried out in Japan and involved 490 previously treated NSCLC patients unselected by *EGFR* mutations. MST were 14.0 and 11.5 months for the gefitinib and docetaxel groups, respectively, and the HR was 1.12 (95 % CI: 0.89–1.40). Thus, the study did not demonstrate noninferiority of gefitinib to docetaxel. The potency of gefitinib in unselected patients with NSCLC is considered to be controversial.

### Selection by background

**Fig. 1:**
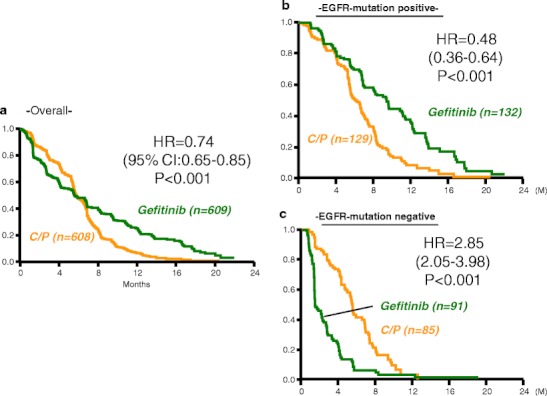
Progression-free survival in IPASS. **a** Kaplan–Meier curves of PFS for Asian patients treated with gefitinib or carboplatin plus paclitaxel who had pulmonary adenocarcinoma and who were light or nonsmokers. **b** and **c** show PFS for patients with or without *EGFR* mutations treated with gefitinib or carboplatin plus paclitaxel, respectively, in subset analyses. [14]

In preplanned subgroup analyses of the ISEL trial mentioned above [20], gefitinib was shown to extend survival in Asian patients (MST: 9.5 months vs. 5.5 months, HR = 0.66, *p* = 0.01). In addition, covariate analyses of demographic subsets among patients of Asian origin treated with gefitinib showed a survival advantage (HR < 1) across never-smokers (HR, 0.37; *p* = 0.0004) and adenocarcinoma patients (HR, 0.54; *p* = 0.0028). Therefore, in March 2006, the Iressa® Pan-Asia Study (IPASS) was initiated to investigate the effectiveness of first-line gefitinib in previously untreated patients in East Asia who had advanced pulmonary adenocarcinoma and who were light or nonsmokers [14]. The IPASS included 1,217 NSCLC patients selected by backgrounds and compared gefitinib therapy with carboplatin (CBDCA) + paclitaxel (PTX) therapy as a first-line treatment. As to PFS, which was the primary endpoint of this study, the HR was 0.741 (95 % CI: 0.651–0.845), and it was reported that the outcome was significantly better in the gefitinib group. However, since the survival curves for the two groups crossed each other, it was difficult to interpret the value of HR (Fig. 1a). Because Cox analysis should be used in cases having a constant relationship between HR and time [21], this could not be used when the curves crossed each other. For example, PFS of gefitinib was better, the same, or worse than that of CDBCA + PTX at 12, 6, or 3 months, respectively (Fig. 1a).

Although the result at the primary endpoint in the IPASS was inconclusive, the importance of the IPASS report is demonstrated in its subset analyses [14]. Among 1,217 patients enrolled, an *EGFR* mutation test (amplification mutation refractory system) was performed on tumor samples from 437 patients (36 %). In this analysis, the crossing of the survival curves seen in Fig. 1a disappeared (Fig. 1b, c). In the subgroup of 261 patients who were positive for *EGFR* mutation, PFS was significantly longer among those who received gefitinib than among those who received CBDCA–PTX (HR = 0.48; *P* < 0.001), whereas in the subgroup of 176 patients who were negative for the mutation, PFS was significantly longer among those who received CBDCA–PTX (HR = 2.85; *P* < 0.001). Thus, the critical message was that there was no indication for gefitinib in patients who were negative for the *EGFR* mutation.

**Fig. 2:**
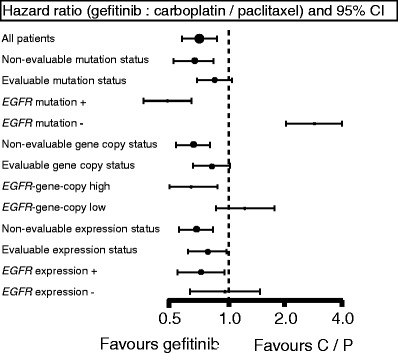
Biomarker for gefitinib. In comparing *EGFR* mutation, *EGFR* gene copy number, and *EGFR* expression status, *EGFR* mutation is the best biomarker for gefitinib. [22]

In addition to the *EGFR* mutation test described above, the biomarkers analyzed in IPASS were *EGFR* gene copy number (fluorescent in situ hybridization (FISH)), and EGFR protein expression (immunohistochemistry) [22]. PFS was significantly longer with gefitinib in patients whose tumors had both high *EGFR* gene copy number and *EGFR* mutation (HR, 0.48) but was significantly shorter when a high *EGFR* gene copy number was not accompanied by *EGFR* mutation (HR, 3.85) (Fig. 2). Among the three biomarkers, *EGFR* mutations are the strongest predictive biomarker for PFS and tumor response to first-line gefitinib vs. CBDCA + PTX. Selection by backgrounds, Asian origin, adenocarcinoma histology, and light or nonsmoking resulted in an *EGFR* mutation-rich population at a rate of 60 % (261 EGFR-mutated patients/437 patients evaluated). Thus, if the strategy of selection by backgrounds is employed, there should be a 40 % risk associated with TKI treatment for patients without *EGFR* mutations.

### Selection by *EGFR* mutations

Since 2004 when the pivotal studies reported on the relationship between EGFR mutations and TKI sensitivity, multiple phase II studies have confirmed a striking response to EGFR-TKIs in this population in Japan [23–29]. A combined analysis employing these phase II studies, named *I*RESSA *C*ombined *A*nalysis of the *M*utation *P*ositives (I-CAMP) study, indicated longer PFS with gefitinib than with standard chemotherapy [30]. In March 2006, at the same time that the IPASS study started, two phase III trials, the North East Japan (NEJ) 002 study and the West Japan Thoracic Oncology Group (WJTOG) 3405 [16, 17], were initiated, which compared gefitinib with standard chemotherapy in first-line treatment for *EGFR*-mutated NSCLC (Table 2). NEJ 002 first confirmed as the primary endpoint that PFS in the gefitinib group was significantly longer than that in the CBDCA plus PTX group (10.8 months vs. 5.4 months, HR = 0.30, *P* < 0.001) [15, 16]. In WJTOG3405, the gefitinib group also had significantly longer PFS compared with the cisplatin plus docetaxel goup, with a median PFS of 9.2 months vs. 6.3 months (HR 0.489, *p* < 0.0001) [17]. In order to evaluate erlotinib further, the phase III OPTIMAL study [18] was initiated in August 2008. It compared the PFS of erlotinib with gemcitabine plus CBDCA in the first-line treatment of Chinese patients with advanced *EGFR* mutation-positive NSCLC. The median PFS was significantly longer in erlotinib-treated patients than in those on chemotherapy (13.1 vs. 4.6 months; HR = 0.16; *p* < 0.0001). In another phase III study, EURTAC [19], started in February 2007, PFS with erlotinib was compared with standard chemotherapy for first-line treatment of European patients with advanced *EGFR* mutation-positive NSCLC. The preplanned interim analysis showed that the median PFS was 9 . 7 months in the erlotinib group, compared with 5.2 months in the standard chemotherapy group (HR = 0 . 37; *p* < 0 . 0001).

**Table 2:** Phase III studies of TKI for *EGFR*-mutated patients

Trial	Arm	Number	RR	PFS	OS	Ref.
NEJ 002	Gefitinib	114	74 %	10.8 m	27.7 m	NEJM (2010)
	CbPXL	110	31 %	5.4 m	26.6 m	OS: Ann Oncol. (in press)
				HR = 0.30*	HR = 0.89	QOL: Oncologist (2012)
WJTOG 3405	Gefitinib	86	62 %	9.2 m	36 m	Lancet Oncol (2010)
	CisDTX	86	32 %	6.3 m	39 m	OS: ASCO (2012)
				HR = 0.49*	HR = 1.19	
OPTIMAL	Erlotinib	83	83 %	13.1 m	NR	Lancet Oncol (2011)
	CbGEM	82	36 %	4.6 m	NR	QOL: ASCO (2012)
				HR = 0.16*		
EURTAC	Erlotinib	86	58 %	9.7 m	NR	Lancet Oncol (2012)
	Pt doublet	87	15 %	5.2 m	NR	
				HR = 0.37*		

*shows a significant difference between arms

OS was retrospectively compared between advanced NSCLC patients with sensitive *EGFR* mutations who began first-line systemic therapy before and after gefitinib approval in Japan (January 1999–July 2001 and July 2002–December 2004, respectively) [31]. In 136 (41 %) of the 330 patients treated at the National Cancer Center Hospital of Japan, although no significant survival improvement was observed in patients without *EGFR* mutations (MST: 13.2 vs. 10.4 months, respectively; *P* = 0.13), OS was significantly longer among the *EGFR*-mutant patients treated after gefitinib approval compared with the OS of patients treated before gefitinib approval (MST: 27.2 vs. 13.6 months, respectively; *P* < 0.001). However, a combined analysis of ICAMP and a post hoc analysis of IPASS suggested identical survival of patients on gefitinib and chemotherapy in first-line treatment for *EGFR*-mutated patients [30, 32].^.^Furthermore, a secondary endpoint of both NEJ 002 [33] and WJTOG3405 [34] prospectively showed identical OS between gefitinib and chemotherapy in first-line treatment of NSCLC patients harboring sensitive *EGFR* mutations (Table 2), although OS data from OPTIMAL and EURTAC are immature at the present time. It must be explained that in almost all of the patients who were treated with first-line chemotherapy in NEJ 002 and WJTOG 3405, a crossover treatment with gefitinib was undertaken. Therefore, from the viewpoint of OS, the effect of gefitinib is additive to that of chemotherapy, indicating that both first-line and second-line gefitinib are acceptable.

When OS is identical between two arms, improvement in quality of life (QoL) and disease-related symptoms are among the key goals in the treatment of NSCLC. IPASS reported better QoL in *EGFR*-mutated patients treated with gefitinib than in those treated with CBDCA + PTX, but this analysis was a post hoc estimation [35]. With the exception of WJTOG3405, the other three trials listed in Table 2 prospectively investigated QoL of NSCLC patients with sensitive *EGFR* mutations who were treated with EGFR-TKI or standard chemotherapy, and NEJ 002 and OPTIMAL have presented the results [36, 37]. In NEJ 002, patients’ QoL was assessed weekly using the Care Notebook [38], and the primary endpoint of the QoL analysis was time to deterioration from baseline on each of the physical, mental, and life well-being QoL scales. Kaplan–Meier probability curves and logrank tests showed that time to defined deterioration in physical and life well-being significantly favored gefitinib over chemotherapy (HR = 0.34; *p* < 0.0001 and HR, 0.43; *p* < 0.0001, respectively); this indicated that QoL was maintained much longer in patients treated with gefitinib than in those treated with standard chemotherapy [36]. In OPTIMAL, the Functional Assessment of Cancer Therapy (FACT) measuring system showed that compared with the gemcitabine/CBDCA group, the erlotinib group had a clinically relevant improvement in QoL, as assessed by scores on the FACT-L (73 % vs. 29.6 %; odds ratio (OR) = 6.9; *p* < 0.0001), the LCSS (75.7 % vs. 31.5 %; OR = 6.77; *p* < 0.0001), and the TOI (71.6 % vs. 24.1 %; OR = 7.79; *p* < 0.0001) [37]. These QoL results conclusively indicate that EGFR-TKI should be considered as the standard first-line therapy for advanced EGFR-mutated NSCLC despite the lack of survival advantage.

### EGFR-TKIs for *EGFR*-mutated patients with poor performance status and advanced age

**Fig. 3:**
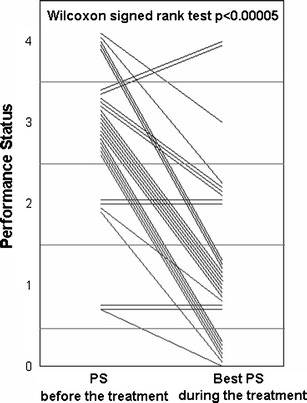
Performance status (PS) improvement by gefitinib in the NEJ 001 Study. Each *line* shows changes of PS in a patient. [39]

The multicenter phase II NEJ 001 study was undertaken to investigate the efficacy and feasibility of gefitinib treatment for advanced NSCLC patients harboring *EGFR* mutations but who were ineligible for chemotherapy due to poor performance status (PS) [39]. The overall response rate was 66 %, and median PFS and MST were 6.5 months and 17.8 months, respectively. PS improvement rate was 79 % (*p* < 0.00005); in particular, 68 % of the 22 patients improved from PS ≥3 at baseline to PS 0 or 1. (Fig. 3) Thus, the “Lazarus Response” was observed in treatment-naïve, poor PS patients with NSCLC and *EGFR* mutations [40]. In patients with sensitive *EGFR* mutations but with extremely poor PS (suspected MST less than 4 months with BSC), the difference in benefit with or without gefitinib treatment was so marked that a randomized phase III study to compare gefitinib to BSC alone may not be justified. This was the first occasion on which changes in treatment guidelines were provoked by a phase II study of NSCLC. Since previously there has been no standard treatment for these patients with short life expectancy other than BSC, examination of *EGFR* mutations as a biomarker is also strongly recommended in this patient population.

In regard to so-called “fit” elderly patients harboring *EGFR* mutations, the NEJ 003 phase II study [41] investigated patients with chemotherapy-naïve history, a median age of 80 years (range: 75–87 years), and PS 0–1, who were treated with gefitinib as a first-line treatment. The response rate was 74 %, and the median PFS and OS were 12.3 months and 33.8 months, respectively. Considering its strong antitumor activity and mild toxicity, first-line gefitinib may be preferable to standard chemotherapy in this population. However, a phase III study comparing gefitinib to standard chemotherapy may be needed to provide the final evidence of benefit in advanced *EGFR*-mutated “fit” elderly patients.

Tarceva Lung cancer Survival Treatment (TRUST) [42] was an open-label, phase IV study of unselected patients with advanced NSCLC. In a subpopulation of elderly patients (≥70 years) receiving first-line erlotinib (*n* = 485) in TRUST [43], the disease control rate was 79 %, median PFS was 4.57 months, and MST was 7.29 months. A total of 87 subpopulation patients (18 %) had an erlotinib-related adverse event (AE); 4 % had a ≥3 grade erlotinib-related AE. Erlotinib was effective and well-tolerated and may be considered for unselected, elderly patients with advanced NSCLC who are unsuitable for standard first-line chemotherapy or radiotherapy. However, there have been few prospective studies of erlotinib in advanced, *EGFR*-mutated, “fit” elderly patients.

## *EGFR* mutation tests

Direct sequencing of *EGFR* requires histology obtained by operation. The NEJ 001, NEJ 002, and NEJ 003 series all used the same *EGFR* mutation test, the peptide nucleic acid-locked nucleic acid polymerase chain reaction clamp (PNA LNA PCR clamp) [44–46]. This is a technological innovation that can make not only tissue-based assessment but also cytology-based assessment of *EGFR* mutations. Briefly, genomic DNA fragments surrounding mutation hot spots of the *EGFR* gene are amplified by PCR in the presence of a clamp primer synthesized from PNA with a wild-type sequence. This leads to preferential amplification of the mutant sequence, which is detected by a fluorescent primer that incorporates LNA to increase specificity. As a result, a mutant *EGFR* sequence is detected in the presence of a 100-fold wild-type sequence. Thus, by the PNA LNA PCR clamp, a small number of *EGFR* mutation-positive cancer cells are detected within 3 h. The sensitivity and specificity of the PNA-LNA PCR clamp were 97 % and 100 %, respectively [46]. Therefore, *EGFR* testing by the PNA LNA PCR clamp was possible in patients with extremely poor PS and of advanced age.

In 2012, the performance, sensitivity, and concordance among five *EGFR* tests of PCR-Invader®, PNA LNA PCR clamp, direct sequencing, Cycleave™, and Scorpion Amplification Refractory Mutation System (ARMS)® were reported [47]. All tests, except direct sequencing, detected mutation types at ≥1 % mutant DNA. Analysis success rates were 91.4–100 %, and interassay concordance rates of successfully analyzed samples were 94.3–100 %. It was concluded that cytology-derived DNA is a viable alternative to formalin-fixed paraffin-embedded (FFPE) tissue samples for analyzing *EGFR* mutations.

It was clarified that frequencies of *EGFR*-mutated NSCLC patients are approximately 31 % and 16.6 % in Japan and Europe, respectively [46, 48]. In Japan, approximately 50,000 patients were newly diagnosed as NSCLC in 1 year. In 2011, approximately 48,000 tests for *EGFR* mutations were carried out under national health insurance, indicating that most patients with NSCLC were screened in Japan. Under circumstances where *EGFR* mutations, *EML4-ALK* fusion gene, and *RET* fusion genes should be tested, routine screening for all of these will be required when making diagnosis of NSCLC.
